# The Role of Autophagy in Eye Diseases

**DOI:** 10.3390/life11030189

**Published:** 2021-02-27

**Authors:** José A. Fernández-Albarral, Esther de Julián-López, Carmen Soler-Domínguez, Rosa de Hoz, Inés López-Cuenca, Elena Salobrar-García, José M. Ramírez, María D. Pinazo-Durán, Juan J. Salazar, Ana I. Ramírez

**Affiliations:** 1 Instituto de Investigaciones Oftalmológicas Ramón Castroviejo, Universidad Complutense de Madrid (UCM), 28040 Madrid, Spain; joseaf08@ucm.es (J.A.F.-A.); estherdejul@yahoo.es (E.d.J.-L.); mcsoldo@hotmail.com (C.S.-D.); rdehoz@med.ucm.es (R.d.H.); inelopez@ucm.es (I.L.-C.); elenasalobrar@med.ucm.es (E.S.-G.); ramirezs@med.ucm.es (J.M.R.); 2 OFTARED: Spanish net of Ophthalmic Pathology, Institute of Health Carlos III, 28029 Madrid, Spain; dolores.pinazo@uv.es; 3 Departamento de Inmunología, Facultad de Óptica y Optometría, Oftalmología y ORL, UCM, 28037 Madrid, Spain; 4 Departamento de Inmunología, Facultad de Medicina, Oftalmología y ORL. UCM, 28040 Madrid, Spain; 5 Ophthalmic Research Unit “Santiago Grisolía”-FISABIO and Cellular and Molecular Ophthalmobiology Unit, University of Valencia, 46017 Valencia, Spain

**Keywords:** autophagy, ocular pathology, glaucoma, cataract, diabetic retinopathy, AMD

## Abstract

Autophagy is a catabolic process that ensures homeostasis in the cells of our organism. It plays a crucial role in protecting eye cells against oxidative damage and external stress factors. Ocular pathologies of high incidence, such as age-related macular degeneration, cataracts, glaucoma, and diabetic retinopathy are of multifactorial origin and are associated with genetic, environmental factors, age, and oxidative stress, among others; the latter factor is one of the most influential in ocular diseases, directly affecting the processes of autophagy activity. Alteration of the normal functioning of autophagy processes can interrupt organelle turnover, leading to the accumulation of cellular debris and causing physiological dysfunction of the eye. The aim of this study is to review research on the role of autophagy processes in the main ocular pathologies, which have a high incidence and result in high costs for the health system. Considering the role of autophagy processes in cell homeostasis and cell viability, the control and modulation of autophagy processes in ocular pathologies could constitute a new therapeutic approach.

## 1. Introduction

Autophagy, a catabolic process that is conserved in the evolutionary process, involves the degradation and recycling of cellular components to maintain the cells’ interior under appropriate conditions for their survival [1]. The process of autophagy is a double-edged sword, as it is involved in both cell death and cell survival [1,2]. Through digestion, autophagy can eliminate debris that may be toxic, causing cell death. It can also digest cellular organelles, which are altered for subsequent recycling [3]. Autophagy also removes defective or aggregated proteins and lipid droplets from the cytoplasm via double membrane vesicles that are transported to and fused with lysosomes for enzymatic degradation [4]. After the digestion process, metabolites that can serve as the basis for the synthesis of new organelles or essential molecules for the cell are generated.

Various stress conditions, such as infections, ionizing radiation, hypoxia, and even chemotherapeutics, can cause the induction of autophagy [5]. Yoshinori Oshumi won the Nobel Prize in 2016 for his research work on autophagy processes. In the early 1990s, he identified genes related to autophagy processes in yeast (*Saccharomyces cerevisiae*) [6], subsequently characterizing the proteins encoded by these genes. He later conducted similar research in mammals [7]. Since the beginning of the 21st century, this catabolic process has been one of the main targets in cellular research in order to study some types of cancer, metabolic, inflammatory, and neurodegenerative diseases as well as cellular aging [1]. In addition, it is known that alterations in autophagy processes are involved in the development of certain ocular pathologies; therefore, treatments focused on the regulation of autophagy processes could offer a therapeutic alternative for the treatment of ophthalmologic pathologies [8].

## 2. Overview of Autophagy

### 2.1. Autophagy Types

**Figure 1:**
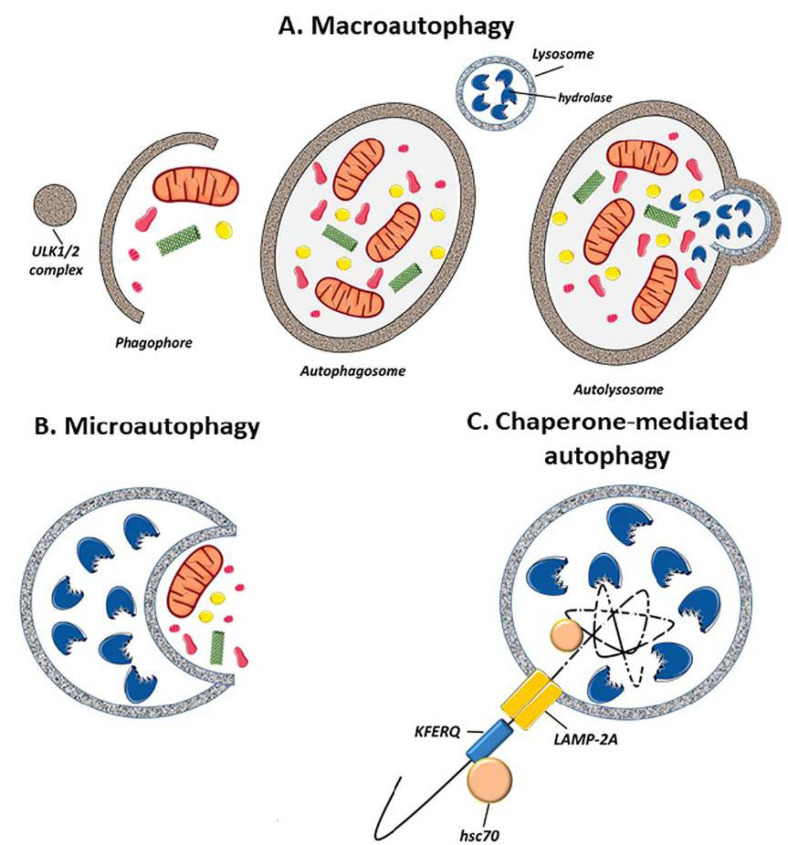
Schematic representation of the different types of autophagy. (**A**) Macro-autophagy: the material to be degraded is enclosed in a double-membraned cytosolic vesicle called the autophagosome. (**B**) Micro-autophagy: cytosol components are enclosed by lysosomes through membrane invaginations. (**C**) Chaperone-mediated autophagy: proteins are detected by the lysosome membrane through a chaperone called hsc70. This complex binds with lysosome-associated membrane protein 2 (LAMP-2A) and is introduced into the lysosome. (Created in part with smart.servier.com, accessed date 20 February 2021).

Autophagy can be classified into three different types: macro-autophagy, micro-autophagy, or chaperone-mediated autophagy (CMA) (Figure 1). The difference between the three depends on how the components to be degraded are transferred to the lysosomes:

(i) Macro-autophagy: The process of macro-autophagy is important in physiological processes such as development, aging, and apoptosis, as well as in pathologies including cancer, infections, and degenerative, metabolic, and inflammatory diseases, among others. The cytoplasmic material is captured by double membrane vesicles (autophagosomes) and transported to the lysosomes for degradation. When autophagosomes fuse with lysosomes, the resulting vesicles (single membrane vesicles) are called autolysosomes [9] (Figure 1A). 

(ii) Micro-autophagy: The lysosomal membrane invaginates to envelop small portions of the material to be degraded, introducing them into the lysosome. [10] (Figure 1B).

(iii) Chaperone-mediated autophagy: this type of autophagy is only observed in mammals. Soluble proteins are incorporated into the lysosome by a transporter located in the lysosome membrane. The transporter is a chaperone complex of the 70 KDa heat shock protein family (hsp70) that recognizes the specific amino acid sequence signal in proteins: KFERQ (pentapeptide: Lysine, Phenylalanine, Glutamic Acid, Arginine and Glutamine). The protein is then translocated into the lysosome. This type of autophagy does not affect organelles or other macromolecules [11] (Figure 1C). 

Autophagy can be selective or non-selective [12].

(i) Selective autophagy: this can achieve the specific removal of harmful substances, cellular organelles, and specific proteins [13]. These components can be recognized by specific receptors. The three types of autophagy mentioned above can act in the following way [14]. There are different nomenclatures for the elimination of different types of organelles, such as "mitophagy" for the elimination of mitochondria. This is very important because its alteration can be related to some neurodegenerative diseases, some of them with ocular involvement [9]. 

(ii) Non-selective autophagy: this is a survival mechanism that is activated by the stress caused by the absence of nutrients. It serves to randomly recycle cellular components and compensate for this deficit. It can be used through both macrophagy and microphagy [15]. 

### 2.2. Regulation of Autophagy: Signaling Pathways

**Figure 2:**
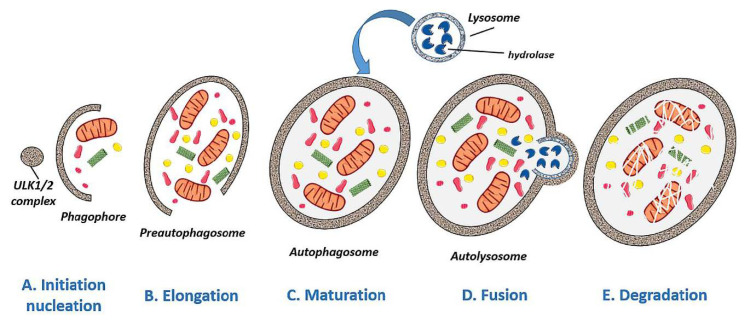
Schematic representation of the different phases of autolysosome formation. (**A**) Initiation/nucleation: a deficiency of nutrients induces autophagy and with it the formation of an insulating membrane to envelop damaged proteins and organelles. (**B**,**C**) Elongation and maturation: the phagophore elongates its double membrane to become a fully-closed mature autophagosome. (**D**,**E**) Fusion and degradation: the autophagosome fuses with a lysosome to form an autolysosome, digesting the material by enzymes (lipases and proteases). (Created in part with smart.servier.com, accessed date 20 February 2021).

Autophagy can be divided into several phases: initiation/nucleation, elongation, maturation, fusion, and degradation. A nutrient deficiency induces the initiation or nucleation of autophagy and with it the formation of an insulating membrane that envelops damaged proteins and organelles (Figure 2A). During elongation, the double membrane of the phagophore extends and eventually closes around the contents to be degraded (Figure 2B), becoming a mature autophagosome (Figure 2C). Finally, the mature autophagosome will fuse with a lysosome to form an autolysosome (Figure 2D) and degrade its contents by proteases and lipases [9] (Figure 2E). 

The mTOR (mammalian Target of Rapamycin) complex is the most important regulator and prevents the initiation of the autophagy pathway by inhibiting the serine/threonine kinase ULK1 when growth factors and nutrients are abundant [16,17]. When nutrients are scarce, the ATP level is greatly reduced, causing the ratio of ATP to AMP to decompensate. This decompensation leads to the activation of AMPK (AMP-activated protein kinase) and, subsequently, to the phosphorylation of ULK1, initiating autophagy [18]. Next, the ULK1 complex binds to membranes containing autophagy-related protein 9 (ATG9), followed by ULK1-dependent phosphorylation of ATG9, thus forming the phagophore [19]. Vesicle nucleation is then initiated by phospholipid incorporation, and a multiprotein complex with phosphatidylinositol 3-kinase (PI3K) activity is recruited. For elongation, the vacuolar protein VPS34 synthesizes phosphatidylinositol 3-phosphate (PI3P) [20]. Subsequently, the autophagosome membranes bind to PI3P-binding ATG proteins and beta-transducin (WD) repeat protein elements that interact with the phosphoinositide (WIPI) family, resulting in the phagosome membranes being completely closed [21]. This mature autophagosome can move and fuse with lysosomes, creating an autolysosome. The contents are then degraded by lysosomal hydrolases to produce energy or synthesize new structures [22]. mTOR signaling (the most important autophagic regulator) is inhibited by p53 and ATG (autophagy-related gene) proteins [23]. Another signaling pathway that regulates autophagy is eukaryotic initiation factor 2α (eIF2α). This factor can be activated in response to endoplasmic reticulum stress, nutrient deficiency, and the presence of double-stranded RNA [24].

### 2.3. Physiological Functions of Autophagy

Many factors are capable of inducing the autophagy process, including oxygen deprivation, nutrient deficiency, or infectious processes. Therefore, it is necessary for this process to be well regulated to provide correct cellular and tissue homeostasis [9]. Autophagy is involved in processes that are essential for cell survival, as it is able to remove altered cellular organelles, eliminate viruses and bacteria from cells, and prevent the accumulation of altered proteins [25,26]. In fact, it has been shown that if two regulators of autophagy are removed, damaged organelles and proteins accumulate, thus increasing the level of reactive oxygen species (ROS) and damaging intracellular components [27,28,29]. In addition, autophagy plays an important role in the maintenance of metabolism and energy levels in both adult and newborn tissues, as has been observed during delivery when the maternal nutrient supply through the placenta is interrupted [30]. In the immune system, autophagy stimulates cytosolic antigen presentation mediated by the major histocompatibility complex class II (MHC II) and affects T-cell and B-cell homeostasis [31]. Other physiological functions of autophagy include protection against oxidative damage to aged membranes and nervous system proteins [32,33], ischemia, hypertension, and response to tumor suppressor genes [34].

Regarding the physiological role of autophagy in the human eye, autophagy also has an important function in cellular homeostasis. Many cells in this organ have a high cellular metabolism rate, a low rate of cell division, or are highly differentiated non-dividing cells [35]. These cells exist in a highly oxidative environment provided by exposure to visible light and ultraviolet radiation which can induce cellular damage [36]. To cope with this oxidative damage, most cell types in the eye make use of autophagy as a cytoprotective method [37]. It has been shown that macro-autophagy is a widespread process that occurs to a greater or lesser degree in different regions of the eye, and selective processes such as mitophagy play fundamental roles in mitochondrial homeostasis [38].

Autophagy-related proteins are highly expressed in the cells of ocular tissues, and in the retina there is greater expression of these proteins during the day [32]. The diurnal variation of autophagosome formation is highly dependent on light; in fact, it has been shown that the concentration of autophagosomes decreases greatly in animals kept in constant darkness [39]. In addition, autophagy processes in retinal pigment epithelium (RPE) cells have been shown to be essential for the functioning of these cells, as they are involved in the phagocytosis of photoreceptor outer segments [40].

### 2.4. Pathological Implications of Autophagy

Autophagy is a process that needs to be under controlled balance. If there is any failure in autophagy pathways, serious pathologies could occur in the organism. An imbalance in autophagy can stimulate the initiation of neurodegenerative diseases such as Huntington’s, Parkinson’s and Alzheimer’s diseases [32,33,41]. Autophagy is essential for cell survival in many solid tumors, as it may be involved in deactivation of apoptosis processes, inducing tumor progression [42,43]. In addition, oncogenes that activate mTOR, and thereby inhibit autophagy, are known to exist [44]. Furthermore, in ocular pathologies, a reduction in autophagy efficiency is associated with diseases such as cataracts, glaucoma, diabetic retinopathy (DR), and age-related macular degeneration (AMD). Therefore, an understanding of the functional roles of autophagy processes in ocular tissues is necessary to allow them to be incorporated into therapeutic strategies for certain ocular pathologies. It is very important to understand the mechanisms and functional roles associated with autophagy in specific cells of the eye before using it as a therapeutic strategy [45].

## 3. Autophagy in Ocular Diseases

Under normal conditions, autophagy plays an important role in the maintenance of cellular homeostasis through the recycling of cytoplasmic proteins and cellular organelles [2]. However, disruption of normal autophagy processes is implicated in the pathophysiology of major ocular diseases [46,47,48,49]. 

### 3.1. Glaucoma

Oxidative stress plays a crucial role in the development of glaucoma by damaging the cells of the trabecular meshwork (TM), which are responsible for regulating the outflow of aqueous humor and maintaining intraocular pressure (IOP) [50]. Therefore, as an individual ages, oxidative damage accumulates, contributing to cell death by apoptosis and the development of glaucoma [51]. To protect themselves from constant oxidative stress and maintain intracellular homeostasis, TM cells activate autophagy to eliminate damaged proteins and organelles. Autophagic activity begins to saturate during the lifetime of an individual, since non-degradable materials accumulate in the lysosome compartment, which consequently causes a decrease in lysosomal activity. This decrease in autophagy leads to progressive dysfunction of TM cells and may contribute to the development of a glaucomatous pathology [52,53,54,55]. Elevated IOP can induce autophagy independently of mTOR to maintain cellular homeostasis in TM cells and cope with the mechanical forces by which they are affected. Porter et al. (2014) [53] conducted an in vitro experiment with pig eyes in which they applied a normal physiological pressure (8 mmHg) to some eyes (control eyes) and a high pressure to others (30 mmHg). Compared with controls, eyes subjected to high pressure showed increased levels of LC3-II (Microtubule-associated protein-1 light chain 3-Type II), a membrane component that is converted from LC3-I (Type I) to initiate autophagosome formation and elongation. In addition, ultrastructural analysis showed the presence of autophagosomes and autolysosomes in TM cells in those eyes subjected to high pressure, a result that was not observed in control eyes. 

During the process of optic nerve cell damage in glaucoma, elevated IOP induces the activation of genes related to autophagosome formation (including *Atg5, Atg7, Atg12*) and autophagy markers such as Beclin-1 and LC3, to increase dendritic autophagic activity [47]. Optineurin has been identified as an autophagy receptor that is involved in cytosolic bacterial removal and the regulation of selective and nonselective autophagy [56,57,58,59,60]. Mutations in the optineurin gene have been associated with normotensive glaucoma [61]. In a study conducted on transgenic mice, overexpression of the most common mutation of the optineurin gene (*OPTN E50K*) was shown to cause a loss of retinal ganglion cells (RGCs) and other cell types [62,63]. In cultured TM cells from donors without an ophthalmic pathology, it was observed that the amount of p62 protein was higher in cells from young individuals than in those from old individuals (over 60 years of age), indicating that autophagy in TM decreases with the aging process [54]. The increased levels of oxidative stress present in glaucoma as a consequence of mitochondrial alteration would be counteracted by mitophagy, which could exert a neuroprotective effect on RGCs [64,65,66]. 

The optic nerve axotomy induces the death of RGCs, a process that occurs in glaucoma, and therefore serves as a model to study the development of this disease. A previous study showed that, five days after mouse optic nerve axotomy, an increase in autophagy occurred and this was associated with autophagosome formation and overexpression of the autophagy regulator Atg5. It was demonstrated that autophagy stimulated RGC survival after axotomy [65]. In this study, mitochondria within autophagosomes were also observed in RGCs. In a similar model of optic nerve axotomy developed in aged autophagy-deficient transgenic mice, it was shown that old individuals were more susceptible to damage compared with young individuals. These aged mice showed alterations in the oxidative stress response and mitochondrial alterations that contributed to increased axonal damage in RGCs [67]. In another study, in a rat model of glaucoma, autophagy was shown to first occur in the dendrites of RGCs rather than in the cell cytoplasm. In addition, autophagosomes located in the dendrites were found to contain organelles, suggesting that mitophagic activity might be active in dendrites [68]. This provides evidence that autophagy could first be activated by cytoprotective stress, but if such stress reaches a limit, apoptosis may be activated. However, in another study, it was shown that, one hour after optic nerve axotomy in rats, autophagosomes appeared in RGC axons. Furthermore, when the autophagy process was inhibited, injury-induced axonal degeneration was enhanced [69]. 

**Figure 3:**
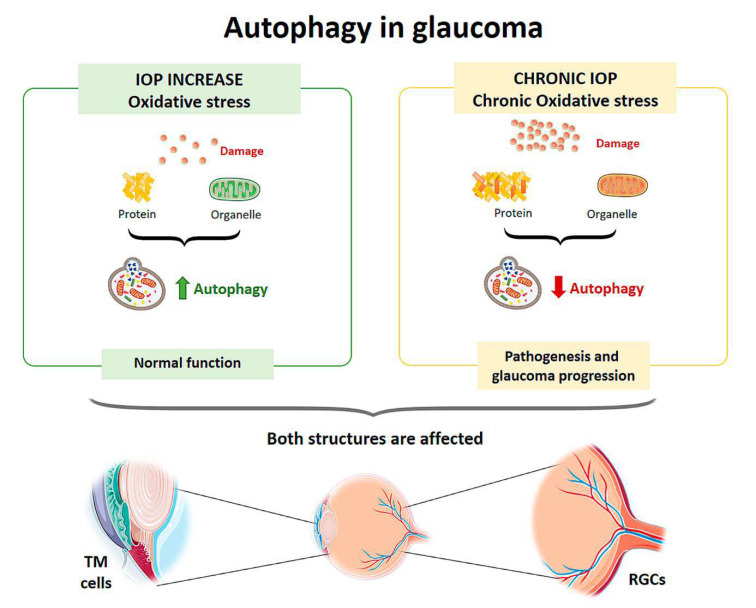
Scheme of the main autophagy changes related to glaucoma. IOP: intraocular pressure; TM cells: trabecular meshwork cells; RGCs: retinal ganglion cells. (Created in part with smart.servier.com, accessed date 20 February 2021).

In cultures of human TM cells derived from glaucomatous eyes in which IOP had been elevated for a long time, when cells were exposed to high oxygen concentrations (40% O_2_), autophagy processes were not activated [55]. Control TM cells from the healthy eye showed an autophagic response to hyperoxia, as there was an upregulation of LC3-II levels, but cells from glaucomatous eyes did not show a variation in this structural protein. Increased LC3 immunoreactivity in the RGC layer as well as LC3-II accumulation was also observed between 6 and 24 h after the transient increase in IOP in an experimental model developed in rats [70,71]. Impairment of the autophagy process by calpain-mediated cleavage of beclin-1 was also demonstrated in this same experimental model [72]. In a transgenic mouse model with altered basal autophagic flux by ablation of the Beclin-1-regulated autophagy-activated molecule (AMBRA-1), increased RGC loss in response to ischemia was observed after a transient increase in IOP [73]. Furthermore, in a transgenic mouse model with impaired autophagic flux and chronic hypertension DBA/2J::GFP-LC3, which express the autophagosome LC3, higher IOP values, further RGC loss, and aggravated axonal impairment were observed compared with the spontaneous ocular hypertensive DBA/2J mouse model [74]. All of these studies demonstrate that autophagy is rapidly activated at the onset of the disease in response to elevated IOP, but if these conditions were chronic autophagic activity would become saturated and cease to function properly. Therefore, the disruption of autophagic flux could be involved in the pathogenesis and progression of glaucoma [75] (Figure 3).

### 3.2. Cataract

**Figure 4:**
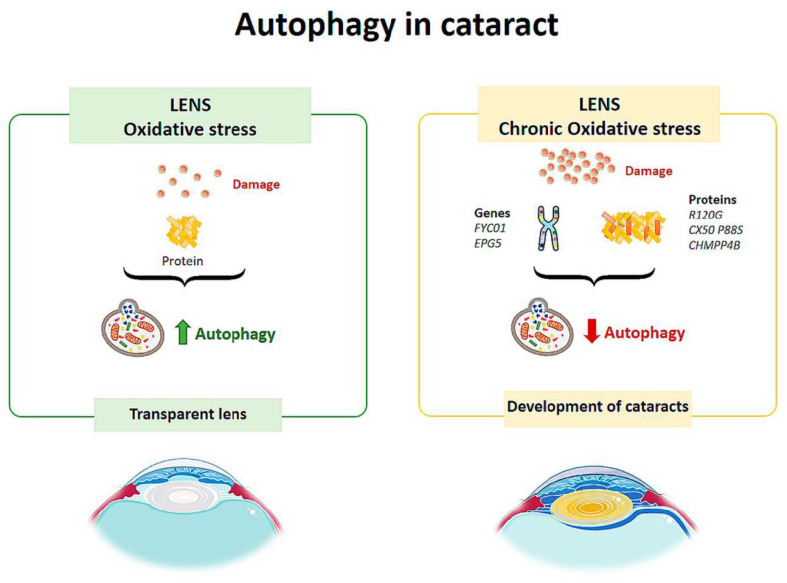
Scheme of the main autophagy changes related to cataract. (Created in part with smart.servier.com, accessed date 20 February 2021).

Autophagic vesicles containing mitochondria or fragments of mitochondria have been observed in human lens fibers. Costello et al. [76] performed a study to confirm the presence of autophagic activity in the lens and demonstrated its presence. Therefore, autophagy helps to maintain the integrity of the cells by contributing to the maintenance of the physical properties of the lens [76] (Figure 4). Disturbances in the elimination of organelles in the cytoplasm of lens fibers increase ROS, altering the homeostasis of the lens, decreasing its transparency and leading to the development of cataracts [77] (Figure 4). 

TBC1D20 (TBC1 domain family member 20) is a key regulator of autophagosome maturation. There is evidence that regulation by TBC1D20 maintains lens transparency. A study conducted on mice tested what would happen if TBC1D20-mediated autophagic flux was altered [78] and found that the animals presented cataracts in the lens nucleus, but the cortex remained transparent. Over time, the pathology progressed, and cataracts completely occupied the lens. In addition, it was observed that the lens fibers were highly degenerated and disorganized, and there was an accumulation of ubiquitin aggregates. Therefore, alteration of the autophagic flux mediated by TBC1D20 resulted in the accumulation of autophagic material in the lens fibers and the formation of cataracts [78]. In another study, Atg5 (autophagy-related protein) knock-out mice showed opacity in the cortical region of the lens after 6–9 months, and by 21 months they had cataracts [79]. The lenses of these mice showed age-dependent cellular disorganization and accumulation of abnormal materials. Accumulation of insoluble p62, ubiquitin aggregates and oxidized proteins was also observed in the lens fibers [79]. These results suggest that autophagy is critical for maintaining intracellular homeostasis in the lens. 

Congenital cataracts are directly related to mutations in several genes. These include recessive mutations in the *FYCO1* gene (FYVE domain and autophagy adaptor coiled-coil domain 1), which codes for a binding protein that regulates adaptor protein (AP) transport [80], a process that is essential for autolysosome formation. There are other mutations in this pathology that may be related to autophagy (Figure 4). Vsp34 is a kinase that plays a role in autophagy, so its loss results in the accumulation of ubiquitin and p62 aggregates in lens cells, affecting tissue transparency [79]. In an inherited cataract model, the R120G mutation in αβ-crystallin (a chaperone that binds to misfolded proteins to prevent their aggregation) results in increases in the LC3-II level and autophagosome size and up-regulates the level of p62 in lens fibers and lens epithelial cells [81]. The CX50 P88S mutation results in a failure of autophagic activity, causing a type of congenital cataract [82]. The *EPG5* gene encodes for a specific autophagic protein. Its deletion in experimental animals mimics the symptoms of Vici syndrome, which is an inherited disease that causes cataracts, among other pathologies. In a 2010 study by Tian et al., it was found that the deletion of *EPG5* in *C. elegans* resulted in the accumulation of autolysosomes that did not degrade their contents [83]; therefore, it was demonstrated that EPG5 is important for the maturation of the autolysosome and that autophagy is involved in this hereditary pathology. Components of the endosomal sorting complex required for transport (ESCRT) are necessary for autophagy and the completion of cytokinesis. The ESCRT subunit called CHMPP4B localizes micronuclei (structures derived from an altered cell division), which are subsequently encircled by lysosomes, and autophagosomes, playing a fundamental role in the process of autophagy [84]. The CHMPP4B mutation is associated with congenital cataracts. This mutation prevents the localization of micronuclei, preventing correct autophagic degradation. Thus, it is suggested as a possible pathway for the development of this pathology. 

### 3.3. Diabetic Retinopathy

In diabetic patients, at the cellular level, hyperglycemia causes the activation of metabolic pathways, resulting in an increase in reactive oxygen species, the appearance of advanced glycation end products, and the release of proinflammatory cytokines, all of which lead to cell death mediated by apoptosis, necroptosis, and autophagy [85]. Therefore, the retinal damage suffered by diabetic patients is related to autophagic events [66].

**Figure 5:**
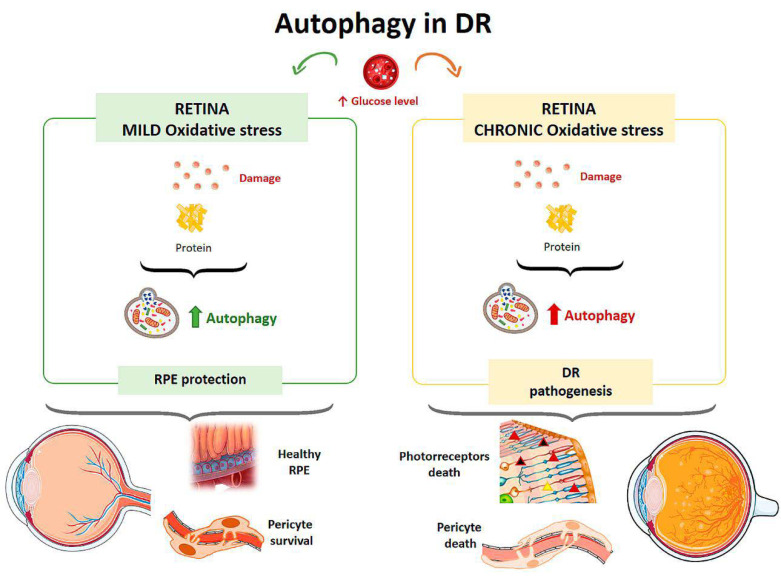
Scheme of the main autophagy changes related to diabetic retinopathy (DR). RPE: retinal pigment epithelium. (Created in part with smart.servier.com, accessed date 20 February 2021).

Autophagy is responsible for removing damaged proteins, providing a defense mechanism against RPE damage, but under conditions of excess metabolic stress autophagic activity is impaired [85] (Figure 5). High glucose levels also affect Müller cells, which are the major source of VEGF (Vascular Endothelial Growth Factor) in DR. In a study on the effect of hyperglycemia on the retina, rat retinal Müller cells were treated with normal or high glucose levels with/without inhibitors and activators for p62 for 24 hours [86]. Cells in contact with high glucose levels responded with increased autophagic activity and endoplasmic reticulum stress associated with increased apoptosis. By inhibiting autophagy in cells treated with high glucose levels, there was an increased number of Müller cells in apoptosis [86]. In a diabetic mouse model, the physiological autophagic flux was observed to be altered, with increased LC3 at the level of the outer plexiform layer and up-regulation of the autophagic proteins Beclin-1 and Atg5 [87]. 

The effect of hyperglycemia on autophagic activity has been demonstrated in cultured human RPE cells [88]. Cultures were incubated in media with a normal glucose concentration (5 mM) and media with a high glucose concentration (30 mM). Cells treated with high glucose levels showed an increased amount of double membrane vacuoles, typical of autophagosomes, at 48 hours after incubation compared with the culture with a normal glucose concentration. In addition, after exposure to hyperglycemic conditions, cells showed higher levels of LC3 in the cytoplasm, indicating an elevated autophagic response. Autophagy induced by high glucose levels was found to be mTOR-dependent but mainly regulated by ROS-mediated endoplasmic reticulum stress signals. Therefore, under a scenario of oxidative stress induced by high glucose levels, autophagy is required to eliminate damaged proteins and provide a mechanism to prevent RPE induced damage [88] (Figure 5). Compared to a control culture, cells that were treated with a high concentration of glucose and were deficient in autophagic processes exhibited a strong reduction in cell viability, suggesting that autophagy plays a protective role for RPE cells in the presence of a DR-common environment [88]. 

The loss of pericytes characteristic of DR leads to increased capillary permeability. Studies on the effect of autophagy performed in a mouse model of diabetes and hypercholesterolemia [89] showed that when low-density lipoproteins (LDL) leaving the vessels accumulate, several cycles of cellular damage are triggered. LDLs were found to cause endoplasmic reticulum stress, oxidative stress, and mitochondrial dysfunction, and these effects induced apoptosis in RPE cells. Autophagy plays a dual role on human retinal capillary pericytes. Thus, autophagy stimulates pericyte survival under mild stress, but if the stress is chronic, excessive autophagic activity results in pericyte necrosis [89] (Figure 5).

In mice in which RD was induced, photoreceptor death occurred before retinal vascular alterations appeared, possibly due to the increase in autophagic activity observed in these cells [87] (Figure 5). The circadian rhythm and diurnal variations in the expression of autophagic proteins are characteristic properties of the retina. In DR, it has been demonstrated that there is an alteration of this biological clock that affects cellular processes such as the regulation of inflammation or lipid metabolism [90,91,92].

### 3.4. Age-Related Macular Degeneration 

Recent studies have shown that RPE cells undergo important pathological changes in AMD, and the alteration of autophagy processes in these cells may play a key role in the development of this pathology [93]. In RPE cells from AMD donors, autophagy levels were found to be decreased with respect to RPE cells from healthy controls [94]. This shows that autophagy is closely related to the pathogenesis of AMD [95].

**Figure 6:**
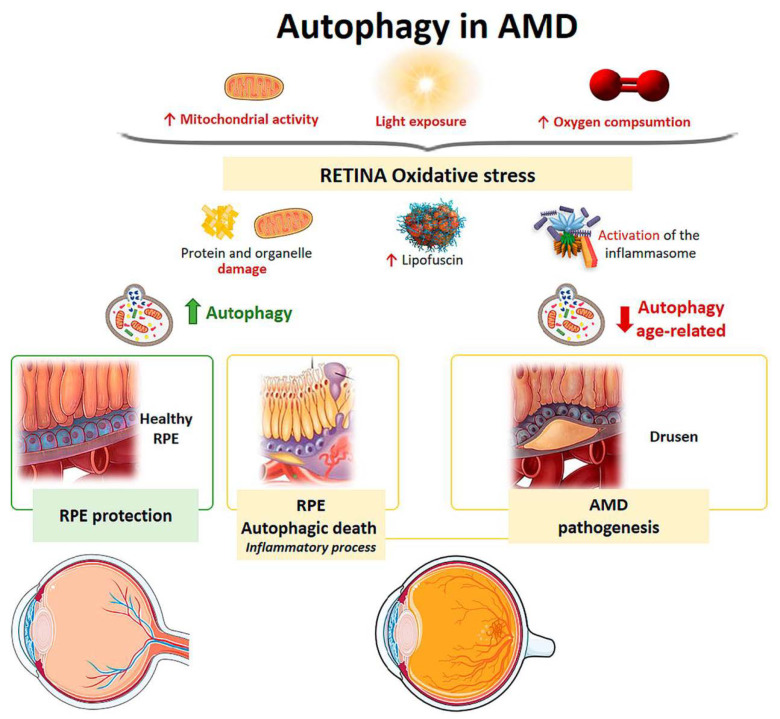
Scheme of the main autophagy changes related to age related macular degeneration (AMD). RPE: retinal pigment epithelium. (Created in part with smart.servier.com, accessed date 20 February 2021).

Mitochondria from human RPE cells exposed to blue light release ROS, resulting in increased damage to the mitochondrial DNA of these cells [96]. Endogenous ROS generation induces autophagy, and it has been observed that, under conditions of oxidative stress, RPE cells in AMD patients generate elevated levels of ROS compared with healthy RPE cells [94]. When oxidative stress occurs continuously over time, mitophagy is activated as a mechanism to protect the cell against this stress. RPE cell dysfunction in AMD donor samples has been linked to mitochondrial DNA injury, and as the disease severity increases, there is greater damage and reduced ability to repair [97]. Therefore, selective removal of damaged mitochondria by autophagy (mitophagy) could be essential for cell survival (Figure 6).

The retina is under intense oxidative stress due to high oxygen consumption, light exposure and high mitochondrial activity leading to the release of ROS in the RPE. To defend themselves, these cells increase the production of anti-oxidants, which decrease with aging, causing severe oxidative damage and progression of AMD. Oxidative stress can be induced by H_2_O_2_. In vitro models have shown that autophagy-associated cell death occurs in ARPE-19 cells and primary human RPE cells by serum deprivation, and H_2_O_2_ (2 h, 1 mM) [98,99]. The phenomenon means, firstly, an induction of a high level of autophagy, which is the cause of cell death, thus autophagic death occurs. Furthermore, these RPE cells undergoing autophagy-associated cell death participate in elimination mechanisms guided by professional and nonprofessional phagocytes and are accompanied by the induction of inflammation. Therefore, this process may be involved in the pathogenesis of AMD [98,99] (Figure 6).

Retinal neovascularization may be related to oxidative stress, pyroptosis (a highly inflammatory form of programmed cell death), and impaired autophagy [100]. Another pathological feature of AMD is the accumulation of lipofuscin in the lysosomes of RPE cells [101]. Lipofuscin is a heterogeneous mixture of lipoproteins that sensitize RPE cells to light exposure and oxidative stress, leading to apoptosis [32,86,102]. A2E (N-retinylidene-N-retinyl-ethanolamine) is a lipofuscin fluorophore that is toxically deposited in the RPE cells of AMD patients. Zhang et al. found that A2E triggers autophagy in RPE cells during the early stages of AMD. Inhibition of autophagy in RPE cells with A2E results in elevated levels of proinflammatory and proangiogenic factors [103]. Therefore, an increase in autophagic activity protects against the harmful effects of A2E.

It has also been observed that reduced autophagic activity is involved in the susceptibility of RPE cells to photo-oxidative damage and, consequently, in the dysfunction of these cells [104,105,106]. Protein aggregates induce the activation of inflammasomes (multiprotein complexes involved in inflammatory processes and pyroptosis) in RPE cells. Increased autophagic activity reduces intracellular debris and causes a decrease in inflammasome generation that could prevent the inflammatory response in AMD [107]. Therefore, if high protein aggregation contributes to inflammasome activation and tissue damage, a decrease in autophagy together with elevated inflammation could be involved in drusen generation [108,109] (Figure 6). 

It has been shown that in RPE cells and photoreceptors of wild-type mice, autophagic levels remain elevated and that exposure to light triggers an autophagic response in these cell types [106]. During the early phase of AMD, autophagic activity acts as protection against damage to organelles and oxidative stress (Figure 6). In human and old mouse AMD samples, LC3, Atg5 were found to be elevated in RPE cells and in drusen [106]. RPE cells from AMD patients have been shown to have impaired mitophagy, resulting in tissue aging [110]. The role of autophagy in the homeostasis of aged RPE cells was demonstrated in mice through a deletion in the gene encoding for the RB1CC1 protein, which is essential for autophagic induction [111]. These mice developed AMD with altered RPE characterized by atrophic patches, subretinal migration of microglia, oxidatively damaged protein deposition, and choroidal neovascularization [111]. RPE degeneration was associated with the loss of neighboring photoreceptors, demonstrating the importance of autophagy in the aging process. βA3/A1-crystallin is a protein that regulates lysosome-mediated degradation in the EPR through the activity of V-ATPase, a proton pump that acidifies the lysosomal lumen through the AKT/mTOR signaling pathway [112,113]. This has been observed in animals deficient in βA3/A1-crystallin, encoded by the Cryba-1 gene, who present a decrease in the lysosomal activity of the EPR, as occurs in patients with AMD. [114]. 

Aging is the main risk factor for developing AMD. In a study on mouse retinas, autophagy levels were compared at different ages [115]. After lysosomal blockade, aged retinas exhibited lipofuscin-laden lysosomes and ubiquitin aggregates characteristic of autophagic impairment. It was shown that chaperone-mediated up-regulation of autophagy would lead to increased lysosome-dependent proteolysis in aged retinas and that there was uncoordination between autophagic pathways in cone-type photoreceptors. The study proposed that this lack of coordination between the different autophagic pathways could be responsible for the specific pattern of visual loss that occurs in aging (Figure 6).

### 3.5. Autophagy as a Therapy in Ocular Pathologies

In recent years, the efficacy of certain drugs that utilize autophagy processes in the treatment of different ocular pathological processes has been proven. These drugs include Rapamycin (RAP), AMPK activator, proteasome inhibitor (MG-132), chloroquine, and hydroxychloroquine, among others [116].

In most ocular diseases, proteolytic and autophagic capacity is attenuated. Therefore, some pharmacological activators of autophagy are being studied as potential therapies for ocular pathologies. 

A drug that acts as a regulator of the autophagy process is Rapamycin, and it is thought to have a potential therapeutic effect in AMD. It acts through the inhibition of mTOR, which leads to an increase in the autophagy process, protecting RPE cells from A2E toxicity and increasing cell viability [103]. In addition to increasing autophagic flux by inhibiting the mTOR pathway, rapamycin treatment, in conjunction with caloric restriction, has been shown to exert a neuroprotective effect on RGCs following transient IOP increase in experimental models of ischemia-reperfusion [73]. This neuroprotective effect of rapamycin has also been observed in animal models of optic nerve axotomy [65] and in models of chronic glaucoma [117].

Resveratrol (natural phenol) and MG-132 (proteasome inhibitor) provide survival stimuli to RPE cells through autophagic induction, so they could be used to prolong the lifetime of RPE cells in AMD [116]. AMPK (AMP-activated protein kinase) is an inhibitor of mTOR, so AMPK-mTOR could be a good target for treating AMD [118]. Metformin regulates AMP kinase activity, which stimulates the initiation of autophagy [119]. Other substances, such as lithium and valproic acid, stimulate the induction of autophagy [120,121]. 

The autophagy process could also be stimulated by using microRNAs. These are endogenous small RNAs that regulate gene expression at the post-transcriptional level. Cai et al. showed that the overexpression of micro-RNA-29 (miR-29) in RPE cells could rescue degenerating cells by enhancing autophagy through inhibiting the activity of mTORC1 [122]. 

Hormones such as 17β-Estradiol and melatonin could promote the autophagy process. Wei et al. 2018 found that 17β-estradiol (βE-2) enhanced autophagy and protected RPE cells from oxidative stress induced by a blue light emitting diode (LED) [123]. Melatonin, a neurohormone derived from tryptophan, which has crucial effects on several systems (circadian rhythm, aging, immune system, and cardiovascular system), as well as being a potent antioxidant, has been shown to positively regulate the autophagy process [95]. It has been shown that in RPE cells exposed to H_2_O_2_ (potent inducer of oxidative damage) and treated with melatonin, there was an increase in autophagy mediated by the up-regulation of LC3-II and Beclin-1 and down-regulation of P62 [124]. 

Some foods with antioxidant properties may help regulate the autophagy process [95]. Intake of fish and nuts can provide n-3 polyunsaturated fatty acids (PUFAs), which may reduce the content of misfolded proteins and inhibit oxidative stress by enhancing autophagy in pathologies such as AMD [125]. In addition, dietary polyphenols found in vegetables, fruits, legumes, or tea can also promote autophagy. This improves the elimination of cellular debris, thereby decreasing oxidative damage, and may help to prevent the development of pathologies such as AMD [126].

On the other hand, excessive autophagic activity may accelerate the development of ocular diseases, so studies should also focus on therapies that inhibit autophagy. 

Chloroquine and hydroxychloroquine suppress autophagy by blocking autophagosome fusion and degradation of autophagic contents. Although some studies have shown that these drugs can be effective and even safe, other studies have shown that these agents can bind to the melanin of the RPE increasing the toxic effects of these drugs. Therefore, treatment with chloroquine and hydroxychloroquine may result in retinal toxicity [116]. 

Sodium trans-hinone IIA sulfonate (STS) has been shown to reduce the expression of the autophagic proteins Atg3, Atg7, and Atg9 by inhibiting vacuole elongation and autophagosome formation. In RPE cells, under oxidative stress conditions, STS activates the mTOR/AKT/PI3K pathway, decreasing autophagy-related cell death [127].

Although the effectiveness of drugs that act on autophagy processes is promising, these drugs nevertheless have some associated problems. They may have undesirable side effects [128,129], as they may have more than one route of action, and they may also have low specificity [128]. Therefore, there is still much to be investigated to eliminate these problems and to obtain safe and effective drugs that can treat ocular pathologies by regulating autophagy processes.

## 4. Conclusions

Autophagic mechanisms, which are essential for the correct homeostasis and cell viability of ocular tissues, may be altered in pathological processes such as glaucoma, diabetic retinopathy, AMD, and cataracts. The regulation and modulation of autophagy could constitute a potential therapy for the treatment of ocular pathologies.

## Data Availability

Not applicable.
